# Exploring the interest of medical students in global health in South Korea: Does taking a global health course matter?

**DOI:** 10.1186/s12909-023-04703-5

**Published:** 2023-10-11

**Authors:** Jayoung Park, Jongnam Hwang, Yeji Lim, Jongho Heo, Woong-Han Kim

**Affiliations:** 1 Department of Human Systems Medicine, Seoul National University College of Medicine, 103 Daehak-ro, Jongno-gu, Seoul, Republic of Korea; 2 Program in Global Surgery and Implementation Science, JW LEE Center for Global Medicine, Seoul National University College of Medicine, Ihwajang-gil 71, Jongno-gu, Seoul, Republic of Korea; 3 Division of Social Welfare & Health Administration, Wonkwang University, 460 Iksandae-ro, Iksan, Jeonbuk Republic of Korea; 4 National Assembly Futures Institute, 1, Uisadang-daero, Yeongdeungpo-gu, Seoul, Republic of Korea; 5 Department of Thoracic and Cardiovascular Surgery, Seoul National University College of Medicine, 103 Daehak-ro, Jongno-gu, Seoul, Republic of Korea; 6 Department of Thoracic and Cardiovascular Surgery, Seoul National University Children’s Hospital, 101, Daehak-ro, Jongno-gu, Seoul, 03080 Republic of Korea

**Keywords:** Global health, Global health education, Medical students, Medical curriculum, Medical education

## Abstract

**Background:**

Prioritizing global health is important for positive health outcomes. Doctors play a pivotal role in addressing health issues that they need to recognize the importance of global health practice. However, medical education in global health is still in its early stages in many countries, including South Korea.

**Methods:**

This study is a quantitative cross-sectional study. Medical students were recruited from all 40 Korean medical schools and data collection was conducted in 2021. Stratified proportional quota sampling was employed as the sampling method. The study examined medical students’ interest in global health as the dependent variable. Independent variables included whether individuals had taken global health courses as part of their medical curriculum, while covariates included student background and institutional factors. Logistic regression was used to determine if taking a global health class was associated with global health interests.

**Results:**

The study included 2450 participants and almost 32% of medical students (n = 781) were interested in global health. Taking global health classes in school was associated with a higher likelihood of developing an interest in global health (OR: 1.29, 95% CI: 1.00-1.67). The likelihood of being interested in global health decreased across the academic year (OR: 0.70, 95% CI: 0.53–0.94). Individuals in graduate entry programs were associated with an interest in global health (OR: 1.32, 95% CI: 1.01–1.72).

**Conclusion:**

This study provides the first comprehensive nationwide assessment of medical students’ perspectives on global health education in South Korea. The findings underscore the importance of early and sustained exposure to global health topics in medical education in fostering interest in global health. These results can serve as valuable evidence for medical educators seeking to integrate global health education into their curricula.

**Supplementary Information:**

The online version contains supplementary material available at 10.1186/s12909-023-04703-5.

## Background

It is essential for those involved in promoting and attaining better health to be interested in global health issues. The fierce competition in our globalized society and limited resources, as well as the many pressing health challenges we face, can sometimes make countries or individuals prioritize their own needs and interests above those of others [1, 2]. However, it is important to recognize that health challenges do not respect national borders or individual interests. Global health emphasizes the importance of universal access to health services and the goal of improving health outcomes for everyone, regardless of their individual circumstances [3–5]. The practice of global health includes cooperation and collaboration in order to address these challenges effectively [6, 7]. Prioritizing global health can have positive ripple effects for many other regions as well in terms of improving health outcomes [8, 9]. As such, to achieve positive health outcomes for all people, individuals, and healthcare professionals, especially doctors who play a critical role in the effort to address health issues and emergencies, need to recognize the importance of collective action and global cooperation in the context of global health.

In light of repeated calls for enhanced global health education in medical training, medical schools have expanded their global health education programs to equip graduates with the essential skills required for global citizenship. Both the World Federation for Medical Education (WFME) and the World Health Organization (WHO) have stressed the significance of integrating global health into medical school curricula [10, 11]. Consequently, numerous medical schools in the UK and the US have taken steps to incorporate global health education into their curriculum, either through elective courses or compulsory elements [12–14]. As a result, the field of global health has gained heightened importance, emphasizing the need for medical students to receive comprehensive training to effectively navigate and contribute to a globalized world.

While global health has become increasingly significant in medical education, many countries, including South Korea, are still in the initial phases of incorporating it comprehensively into their medical curricula [15]. South Korea’s inclusion of global health education is in ongoing development, characterized by a limited range of global health courses and a lack of full integration into the core medical curriculum. The roots of global health in South Korea can be traced back to the Minnesota Project, a partnership between the United States government and South Korea’s medical education system in the 1950s. During this period, South Korea was a developing country with limited resources and access to advanced medical education. Through this collaboration with the University of Minnesota, South Korea had the opportunity to train Korean doctors, gain valuable insights, adopt best practices in healthcare [16, 17]. As a result, South Korea has now transitioned into a donor country, actively participating in the provision of development assistance in medical education and contributing to global health initiatives [18, 19]. This serves as evidence of the positive influence that can be generated through investments in medical education and partnerships with other nations, leading to tangible improvements in global health. Given South Korea’s history of benefiting from global health initiatives as a developing nation, including the transformative Minnesota Project that trained its doctors, it is vital that South Korea prioritizes global health education. Taking a leadership role in global health initiatives and preparing future health professionals to contribute to this field is crucial for South Korea, in light of its past advantages from global health collaborations.

To address this gap, we used a nationally collected survey to explore medical students’ perspective on global health and global health courses provided in the medical school curriculum. The aims of this study were to examine the influence of global health course participation on medical students’ interest in global health and ultimately provide evidence-based recommendations for integrating global health education into medical school curricula.

## Methods

### Data

The Nationwide Survey of Medical Students’ Global Health Exposure study is a national-wide, questionnaire-based cross-sectional survey implemented by the JW LEE Center for Global Medicine at Seoul National University College of Medicine in South Korea.

To have a balanced and representative sample of medical students across medical schools and demographic categories, stratified proportional quota sampling method were used to recruit respondents [20]. First, among the medical students in all the 40 medical schools in Korea, sampling were allocated proportionally based on the number of enrolled students within each school (strata) as per the 2020 university announcement data (available from the Higher Education in Korea website, https://www.academyinfo.go.kr/index.do). Then, the sample was stratified by gender and academic year within each medical school. Participants were selected in the same proportions as population of nationwide medical students recorded on the university announcement data prior to the survey. The sampling strategies ensures to have a nationally representative sampling of medical students across genders (65.0% male and 35.0% female) and academic years.

The survey was collected via online from January 1st 2021 to June 30th 2021 due to COVID-19 restriction on in-person contact. To facilitate sample recruitment, official letters were sent to universities requesting permission to post a survey advertisement on their websites, and the ad was also posted on the website of the Korean Medical Student Association. A total of 2,450 medical students were responded.

### Variables

The survey questionnaire covers the following main topics: demographic information, level of interest, prior global health class experience, medical school information, overseas experience, English proficiency, career aspiration in global health, and whether the medical school curricula include global health. For this study, we chose the following variables:

#### Dependent variable: Medical student’s interest on global health

To assess participant’s interest on global health, we used the question “*How interested are you in global health?*”. Eligible responses on a four-point Likert scale were as follows: “1-very interested”, “2-interested”, “3-not very interested”, or “4-not at all interested”. In accordance with previous literature [21, 22], the scale was subsequently recoded into a dichotomous variable, where scores of 1 or 2 were categorized as “yes,“ and scores of 3 or 4 were categorized as “no.“

#### Independent variable: Global health course participation

Our independent variable of interest was whether individuals took courses on global health as part of a medical curriculum. We used the question “*Have you ever taken global health-related classes while in medical school (either core courses or electives)?*” and the eligible responses were “Yes” or “No”.

#### Covariates

Student background factors (gender, English proficiency, overseas experience, academic year, medical program, career aspiration in global health) and institutional factors (location of medical schools, types of medical schools, school curricula including global health course) based on prior literature [13, 14, 23]. In this context, South Korea offers two medical school admission pathways: standard entry and graduate entry. Standard entry is for high school graduates who need to complete six years of study (two years of pre-med and four years of medical course). Graduate entry is for students who have already completed their undergraduate studies and only need to complete four years of medical school.

### Statistical analysis

Logistic regression was used to determine the odds ratios for the associations between taking a global health class as part of a medical curriculum and the participant’s interest in global health. The socio-demographic variables, medical school and academic curricula-related variables were considered as potential confounders in this study. Possible association between each of the predictor variables, each of the other independent variables and the outcome variable (medical student’s interest in global health) were assessed. Statistical analyses were done using Stata 17 (StataCorp, 2021). The results are presented as odds ratio (OR) and 95% CI.

## Results

The descriptive characteristics of the 2450 participants are shown in Table 1. A total of 31.9% (n = 781) of medical students reported an interest in global health. Among individuals who are interested in global health, first year medical students indicated significantly more interested in global health than those in upper classes (35.9% in first year, 32.3% in second year, 29.0% in third year, 27.3% in fourth year). Students enrolled to the graduate entry programs were significantly more interested in global health than those admitted through the undergraduate application cycle. Those who intend to pursue a career in global health expressed significantly greater interest in global health (63.0%) than those who do not intend to pursue a career in global health.

**Table 1:** Descriptive characteristics of the survey participants by their interest in global health, The Nationwide Survey of Medical Students’ Global Health Exposure, 2021, Korea (n = 2450)

Variables	Medical students’ interest in global health		
	Yes (n = 781)	No (n = 1669)	p-value^a^
Global health course participation			
No	574 (30.3)	1318 (69.7)	< 0.01*
Yes	207 (37.1)	351 (62.9)	
***Student background factors***			
Gender			
Male	368 (27.8)	955 (72.2)	< 0.01*
Female	413 (36.6)	714 (63.4)	
English proficiency			
Advanced	29 (23.4)	95 (76.6)	< 0.01*
Intermediate	405 (29.4)	970 (70.6)	
Basic	347 (36.5)	604 (63.5)	
Overseas experience			
None	520 (29.8)	1222 (70.2)	< 0.01*
Less than 6 months	123 (37.5)	205 (62.5)	
More than 6 months	138 (36.3)	242 (63.7)	
Academic year			
MD 1st year	290 (35.9)	517 (64.1)	< 0.01*
MD 2nd year	221 (32.3)	464 (67.7)	
MD 3rd year	149 (29.0)	365 (71.0)	
MD 4th year	121 (27.3)	323 (72.7)	
Medical program			
Standard entry	640 (31.0)	1427 (69.0)	0.02*
Graduate entry	141 (36.8)	242 (63.2)	
Career aspiration in global health			
No	374 (20.7)	1430 (79.3)	< 0.01*
Yes	407 (63.0)	239 (37.0)	
***Institutional factors***			
Location of medical schools			
Seoul Capital Area	225 (30.3)	517 (69.7)	0.84
Chungcheong area	141 (33.3)	282 (66.7)	
Honam-Jeju area	149 (33.4)	297 (66.6)	
Daegu-Gyeongbuk-Gangwon area	173 (33.3)	347 (66.7)	
Busan-Gyeongnam area	93 (29.1)	226 (70.9)	
Types of medical school			
Public	231 (32.0)	490 (68.0)	0.91
Private	550 (31.8)	1179 (68.2)	
School curricula on global health			
Yes	124 (36.1)	219 (63.9)	< 0.01*
No	252 (36.7)	434 (63.3)	
Don’t know	405 (28.5)	1016 (71.5)	

*p < 0.05

^a^ For comparison of interest in global health between the “yes” and “no” groups, t-tests and chi-square tests were conducted accordingly.

Table 2 shows the findings for student background and institutional factors influencing medical students’ interest in global health. The findings revealed that taking global health classes in school was associated with a higher likelihood of developing an interest in global health (OR: 1.29, 95% CI: 1.00-1.67). A lower likelihood of being interested in global health was observed across the academic year (OR: 0.70, 95% CI: 0.53–0.94). Individuals in graduate entry programs were associated with developing an interest in global health (OR: 1.32, 95% CI: 1.01–1.72). individuals who had aspirations for a career in global health were significantly more likely to develop an interest in the field (OR: 6.06, 95% CI: 4.95–7.41). In contrast, the data reveals that neither English proficiency nor overseas experience were significant determinants of interest in global health.

**Table 2:** Odds and 95% CIs from logistic regression models examining experience of global health course, student background factors, and institutional factors and an interest in global health among medical students, The Nationwide Survey of Medical Students’ Global Health Exposure, 2021, Korea (n = 2450)

Variables		Medical students’ interest in global health	
		OR	95% CI
*Experience of global health course*			
Global health course participation	No (ref.)		
	Yes	1.29*	(1.00–1.67)
*Student background factors*			
Gender	Male (ref.)		
	Female	1.12	(0.93–1.36)
English proficiency	Advanced (ref.)		
	Intermediate	1.09	(0.69–1.73)
	Basic	1.34	(0.83–2.15)
Overseas experience	None (ref.)		
	Less than 6 months	1.20	(0.91–1.59)
	More than 6 months	1.12	(0.84–1.48)
Academic year	1st year (ref.)		
	2nd year	0.92	(0.72–1.17)
	3rd year	0.71*	(0.54–0.94)
	4th year	0.70*	(0.53–0.94)
Medical program	Standard entry (ref.)		
	Graduate entry	1.32*	(1.01–1.72)
Career aspiration in global health	No (ref.)		
	Yes	6.06*	(4.95–7.41)
*Institutional factors*			
Locations of medical school	Seoul Capital Area (ref.)		
	Chungcheong area	1.05	(0.78–1.42)
	Honam-Jeju area	1.12	(0.84–1.51)
	Daegu-Gyeongbuk-Gangwon area	1.02	(0.78–1.34)
	Busan-Gyeongnam area	0.98	(0.71–1.36)
Types of medical school	Public (ref.)		
	Private	1.06	(0.85–1.33)
School curricula on global health	Yes (ref.)		
	No	1.15	(0.82–1.60)
	Don’t know	0.77	(0.56–1.06)
Constant		0.22*	(0.12–0.41)

Pseudo R^2^ = 0.1356*

*p < 0.05

## Discussion

The study first discovered that the participation in global health classes increases the likelihood of students being interested in global health by 1.29 times compared to those who did not take such class. Secondly, third-year students exhibit a 29% lower likelihood of being interested in global health compared to first-year students. Additionally, fourth-year students show a 30% lower likelihood of being interested in global health than first-year students. Thirdly, medical students who entered medical school as graduate entry display a 1.32 times higher likelihood of being interested in global health compared to those who entered medical school as standard entry.

The primary finding of this study highlights the positive impact of taking global health classes on students’ interest in global health. This underscores the importance of expanding the availability of global health courses in medical schools to cultivate students’ enthusiasm for and involvement in global health activities. Global health education encompasses essential principles such as disease burden and social determinants of health, human rights, health disparities, and ethics, which are crucial for health professionals to address contemporary health problems [14, 24, 25]. Accordingly, global health education equips medical students with critical thinking skills to understand the barriers to healthcare faced by medically underserved populations [23, 26, 27]. Considering the significant influence of medical school experiences on students’ career decisions and practice choices, including their choice of medical specialties and the populations they serve [28], integrating global health education can potentially inspire students to serve underserved populations. Yet, few medical schools in Korea offer global health training as part of their medical education curriculum (Additional file 1). Therefore, institutions should prioritize investment in global education and incorporate relevant courses to help future physicians develop the interest in global health, ultimately producing healthcare professionals who can effectively respond to global health issues affecting marginalized populations.

The study’s second finding emphasizes a decline in interest in global health among third and fourth-year students. This trend may be partially attributed to a shift in students’ focus as they progress through their medical education. As students advance, they tend to become increasingly engrossed in individual studies and specific preparations, such as preparing for the medical licensing examination [29]. In Korea, this shift is even more pronounced. Most fourth-year medical students are on the verge of entering real-world clinical practice, a phase often marked by insufficient classroom and clinical education in global health [30]. This transition is critical in shaping students’ interests. Adding complexity to the issue, research has shown a decline in empathy and idealism among medical students as they advance in their studies [31–33], including one study that specifically noted a decrease in empathy among fourth-year students compared to other grades [34]. These changes often result in reduced motivation to serve underserved communities and a diminished sense of societal responsibility [35, 36]. Considering these factors, the limited exposure to global health topics during this critical phase could play a significant role in the observed decrease in interest among these students.

To address this issue, we propose incorporating experiential learning opportunities, such as global health electives or international rotations, that provide students with firsthand experiences in diverse healthcare settings. By engaging in these experiences, students can enhance their understanding of global health issues and develop crucial cross-cultural communication skills, adaptability, and a stronger sense of social responsibility. It is essential for medical schools to collaborate with global health organizations, non-governmental organizations, and international partners to facilitate clinical rotations, research projects, and community engagement in global health initiatives. Through these collaborations and experiential learning opportunities, medical schools can not only reignite students’ interest in global health but also equip them with the practical skills and knowledge needed to address global health challenges effectively. By providing these opportunities, students can cultivate a broader perspective on healthcare, develop cultural competency, and contribute meaningfully to improving health outcomes in underserved populations worldwide.

The study’s third finding suggests that students in graduate entry programs for medicine are more interested in global health issues than students admitted through standard entry. This finding implies that graduate entry students are more sensitive and interested in issues related to global health. Previous research has shown that standard entry students are motivated by parental expectations, while more graduate entry students are motivated by the need for professional independence and the desire to prevent disease [37]. The diverse range of backgrounds represented in graduate entry students may explain this result, as they are exposed to different fields before receiving medical education [38]. To expand global health knowledge among all medical students, medical schools should consider offering or requiring global health courses for standard entry students during their pre-med coursework.

The results should be viewed within the context of some limitations. Because this study collected data from a cross-sectional survey, it is not possible to determine causality between changes in students’ perspectives and experiences and demographic data. In the future, longitudinal studies should be conducted to analyze how students’ interests change over time. Due to recategorization of certain response options for analysis, the distinctiveness across groups might have been reduced, especially for variables related to medical school locations and ages. Some variable response categories had to be recategorized for analysis, which resulted in reduced distinctiveness across groups, especially for variables relating to medical school locations and ages. Considering this is an online survey, technical issues such as website glitches or slow loading times may occur and prevent the completion of online surveys, making the data incomplete or unusable. Moreover, due to the limited number of schools offering global health as a core course in their medical curriculum, we were unable to stratify the analysis of global health classes as “core” and “elective” courses. This lack of stratification might have influenced our understanding of how compulsory versus optional global health education impacts students’ interests. Further research is crucial to advocate for the development of more core global health courses within medical curricula. Such studies can provide insights into the curriculum’s design, aiming to foster genuine interest and commitment among medical students towards global health challenges. Nevertheless, this is currently the only study of medical students nationwide that has a sufficient sample size for valid conclusions to be drawn.

## Conclusion

This study offers valuable insights into the perspective of Korean medical students on global health education. The findings serve as a strong basis for further exploration into how global health education impacts students’ interests, career paths, and research pursuits. Medical schools can apply these findings to inform the design and implementation of global health courses and programs, enhancing students’ overall competency in this crucial field. Policymakers can also leverage these results to advocate for the integration of global health as an essential element in medical education. Such advocacy has the potential to influence national guidelines and accreditation standards, shaping the future of medical education with regard to global health. Ultimately, this study contributes to our broader understanding of global health education and its potential to transform medical education and enhance global health outcomes.

### Electronic supplementary material

Below is the link to the electronic supplementary material.

Supplementary Material 1

## Data Availability

The datasets used and/or analyzed during the current study available from the corresponding author on reasonable request.
