# Supplementary material for: Exploring the interest of medical students in global health in South Korea: Does taking a global health course matter?
Source: BMC Med Educ. 2023 Oct 11;23:750. doi: 10.1186/s12909-023-04703-5 (PMC10566093; doi:10.1186/s12909-023-04703-5)
Supplement: Supplementary file 1 — Supplementary Material 1 [file 12909_2023_4703_MOESM1_ESM.docx]

| Supplementary Table 1. List of medical schools in South Korea offering global health training as part of their medical education curriculum | | | | |
| --- | --- | --- | --- | --- |
| Institution | **Title of Global Health Course** | **Core/**  **Elective** | **Credit** | **Course term** |
| Kosin University | Medical missionary work and international cooperation | Core | 2 | Med 3YR |
| Seoul National University | Society and Medicine and Community 3 (Global Health) | Core | 3 | Med 2YR |
| Seoul National University | Elective Intermediate Research Course - Global Medicine | Elective | 3 | Med 2YR |
| Seoul National University | Elective Advanced Research Course - Global Medicine | Elective | 3 | Med 4YR |
| Yonsei University | Education (II) (International Cooperation and International Medical Care) | Core | 2 | Med 1YR |
| ** Schools were selected based on the titles of their courses that include terms related to “global” health. Since our participants are medical students, we focused on offering courses to medical students instead of pre-med students.* | | | | |

**Supplementary Table**
